# Comparison of two types of exercises in the treatment of lumbar spinal stenosis

**DOI:** 10.12669/pjms.344.15296

**Published:** 2018

**Authors:** Wenzhi Mu, Yong Shang, Zhuomao Mo, Shujie Tang

**Affiliations:** 1 Wenzhi Mu, Department of Functional Inspection, Yidu Central Hospital of Weifang, Qingzhou, Shandong province, 262500, China; 2 Yong Shang, Department of Orthopaedics, Qingzhou Hospital of Chinese Medicine, Qingzhou, Shandong province, 262500, China; 3 Zhuomao Mo, School of Chinese Medicine, Jinan University, Guangzhou, 510632, China; 4 Shujie Tang, School of Chinese Medicine, Jinan University, Guangzhou, 510632, China

**Keywords:** Conventional exercise, Core stability exercise, Lumbar spinal stenosis (LSS)

## Abstract

**Objective:**

To evaluate the efficacy of core stability exercise versus conventional exercise in the treatment of lumbar spinal stenosis.

**Methods:**

Between January 2014 and May 2017, patients with lumbar spinal stenosis were recruited and divided into group of core stability exercise or conventional exercise randomly. All the patients were treated using middle frequency electrotherapy, in addition to that, the patients in group of core stability exercise were treated using core stability exercise. The patients in group of conventional exercise were treated using conventional exercise. The outcome was evaluated using Japanese Orthopedic Association (JOA) score, self-reported walking capacity and lumbar lordosis angle at baseline and after treatment.

**Results:**

In the current study, sixty-two patients with lumbar spinal stenosis met the inclusion and exclusion criteria, in which 33 patients were included in group of core stability exercise and 29 in group of conventional exercise. After treatment, both Japanese Orthopedic Association scores (p<0.05) and self-reported walking capacity (p<0.05) increased significantly in each group when compared with baseline. The self-reported walking capacity and JOA scores in the group of core stability exercise were significantly higher than those in the conventional exercise group (p<0.05). However, both the intragroup and intergroup comparison of lumbar lordosis presented with no significance (p>0.05).

**Conclusion:**

Core stability exercise presents with better efficacy than conventional exercise in the treatment of lumbar spinal stenosis.

## INTRODUCTION

Lumbar spinal stenosis (LSS) is one of the common spinal disorders in old adults, which affects the life quality of patients, and remains a major cause of morbidity, disability and lost productivity^1^, exerting a heavy burden on social security systems.^2^ The disease can be treated using conservative or surgical modalities.^3^,^4^ Currently, the efficacy of surgical treatments for LSS as compared to nonsurgical modalities remains unclear^3^. Some studies have showed no significant difference in long-terms efficacy between nonsurgical and surgical treatment for LSS^5^, and most scholars advocate that nonsurgical treatment should be the primary option for the disease.

In terms of the nonsurgical managements for LSS, it includes medication, exercises, epidural injections, physiotherapy, lifestyle modification, and some rehabilitative approaches, among which exercises play an important role in the treatment of LSS. In a study of forty-five cases with LSS, Ahmet found that the leg pain and disability score in patients treated using exercises decreased significantly.^6^ In another study of fifty-one cases, Fang found the visual analogue score decreased and Japanese orthopedic association score increased significantly in patients treated using lumbar extension exercise.^7^ Some other studies also drew the similar conclusions^8^,^9^, demonstrating the satisfying effectiveness of exercises in treating LSS.

Moreover, in recent years core stability exercises have been performed widely and scholars suggest that it can provide a positive effect on reducing pain and improving trunk stability to reach an improvement in lumbar function in the rehabilitation of low back pain.^10^ In many fields, core stability exercise presented with more advantages than conventional exercise.^11^ However, which exercise is more favorable for patients with LSS, core stability exercise or conventional exercise? Up to now few studies have been published to evaluate the efficacy of core stability exercise versus conventional exercise for LSS in English literatures.

Therefore, we carried out the current study to compare the efficacy of two types of exercises in the treatment of LSS. The study may help clinicians better make treatment strategies for LSS.

## METHODS

The patients with LSS were recruited to participate in the current study from January 2014 and May 2017.The inclusion criteria included:

1. Patients diagnosed with LSS based on symptoms and MRI.
2. Patients who agreed to participate in the study and signed the informed consent form.

Those patients with cauda equine syndrome, Paget’s disease, severe osteoporosis or metastasis to the vertebrae, significant scoliosis (Cobb angle>25°), previous laminectomy, degenerative or lytic spondylolisthesis or significant instability of lumbar spine were excluded.^2^ The clinical data including age, gender, disease course, and stenosis level were collected and evaluated. This study was approved by the Ethics Committee of our hospital.

The included patients were randomly assigned into group of core stability exercise or conventional exercise, according to random number table. Middle frequency electrotherapy was performed for all patients, 30 minutes each time, once daily for four weeks. In addition, the patients in group of core stability exercise performed core stability exercise including plank, side plank, bridge, and modified push-up^2^, and those in group of conventional exercise performed conventional exercises including sit-up, five-point, four point and three-point lumbar extension exercises^7^. Each movement was carried out ten times, once daily for four weeks in each group. The clinical outcome was evaluated using Japanese Orthopedic Association (JOA) score and self-reported walking capacity at baseline and after treatment. To evaluate the influence of exercise on lumbar lordosis, radiographs of all patients were evaluated to obtain the lumbar lordosis angle before and after treatment, and the angle was defined as the angle subtended by the superior end plateline of L1 and the superior end plate line of S1.^12^ The basic clinical data and outcome measurements between two groups were compared and analyzed.

### Statistical analysis

Statistical analysis was conducted using SPSS21.0 (SPSS Inc., Chicago, IL, USA). The comparisons of baseline clinical data between two groups were carried out using Chi square test or independent sample t-test. The intragroup comparisons of JOA, self-reported walking capacity or lumbar lordosis before and after treatment were performed using paired t test, and the intergroup comparisons using independent sample t-test. A p-value less than 0.05 indicate statistical significance.

## RESULTS

Sixty-two patients with LSS who met the inclusion and exclusion criteria were included in the current study, in which 33 were assigned in group of core stability exercise and 29 in group of conventional exercise. In group of core stability exercise, there were 21 males and 12 females, and conventional exercise group, there were 18 males and 11 females. The baseline clinical characteristics of the two groups are shown in Table-I, there were no significant differences in age, gender, stenosis level, and disease course between the two groups (p>0.05).

**Table-I:** The baseline characteristics of two groups.

Variable	Core stability exercise	Conventional exercise	P-value
Number of patients	33	29	-
Age (years)	55.7±12.8	53.9±13.6	P>0.05
Gender(Male/Female)	21/12	18/11	P>0.05
Stenosis level			P>0.05
One level	15	13	
Two level	13	10	
Three levels	5	6	
Disease course (Month)	69.8±16.5	71.1±15.4	P>0.05

**Fig.1:**
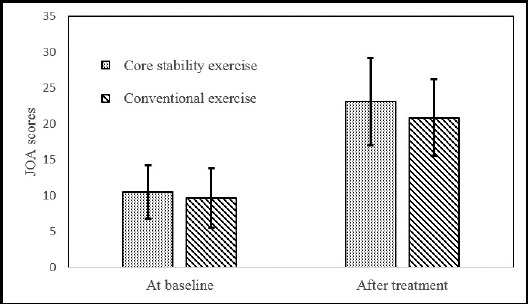
The JOA scores in two groups.

**Fig.2:**
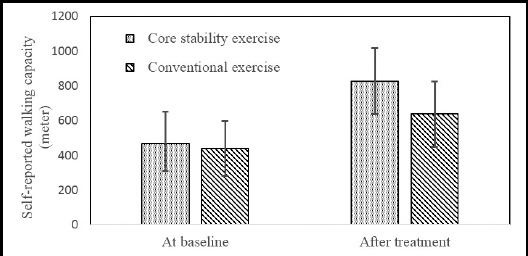
The self-reported walking capacity in two groups.

**Fig.3:**
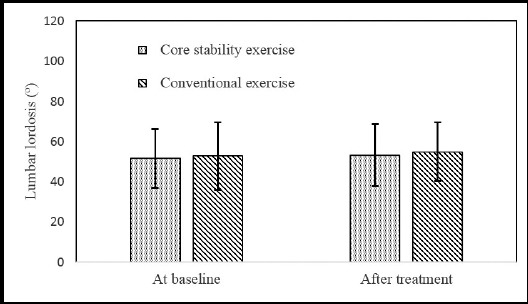
The lumbar lordosis in two groups.

The comparison of JOA, self-reported walking capacity and lumbar lordosis at baseline and after treatment between two groups are shown in Table-II, Fig. 1, 2 and 3. At baseline, there were no significant differences in JOA scores (p>0.05), self-reported walking capacity (p>0.05) and lumbar lordosis (p>0.05) between the two groups. After treatment, both JOA scores (p<0.05) and self-reported walking capacity (p<0.05) increased significantly in each group when compared with baseline. In addition, both the self-reported walking capacity and JOA scores in group of core stability exercise was significantly higher than those in the group of conventional exercise (p<0.05). The mean of lumbar lordosis angle after treatment in both groups was higher than that at baseline, but there was no significant difference (p>0.05). The intergroup comparison of lumbar lordosis angle also showed no significant difference (p>0.05).

**Table-II:** Comparison of JOA, self-reported walking capacity and lumbar lordosis angle between groups.

Outcome measurements	At baseline	After treatment
**JOA scores**		
Core stability exercise	10.5±3.7	23.2±6.1^ab^
Conventional exercise	9.7±4.1	20.9±5.3^a^
**Self-reported walking capacity (meter)**		
Core stability exercise	468±184	825±191^ab^
Conventional exercise	439±159	636±189^a^
**Lumbar lordosis angle**		
Core stability exercise	51.6±14.7	53.1±15.4
Conventional exercise	52.9±16.8	54.9±14.6

Note:

a indicates p<0.05 in intragroup comparison, and

b indicates p<0.05 in intergroup comparison.

## DISCUSSION

In the current study, we compared the efficacy of core stability exercise and conventional exercise in the treatment of LSS. To the best of our knowledge, few studies in this field have been published in English literatures. The study may help clinicians select treatment modalities correctly for patients with LSS.

We found in each group both JOA scores and self-reported walking capacity significantly increased after treatment, demonstrating that two types of exercises together with middle frequency electrotherapy can improve the lumbar function, reduce pain and increase the quality of life in patients with LSS. Middle frequency electrotherapy has effect of analgesia, improving circulation, promoting the absorption of inflammation.^13^ The efficacy of the exercises on LSS may be contributed to its effect on lumbar alignment.^2^ In Toprak’s study, 56 university students were randomly allocated to exercise or control groups, and significant differences were observed for postural pain, thoracic and lumbar curvature, and other parameters in the exercise group between baseline and after treatment.^14^ Kadono’s study also confirmed that stretching exercise can increase lumbar lordosis angle.^15^ Moreover, patients with degenerative disc disease were confirmed to have lower lumbar lordosis and more vertical sacral profiles.^16^ In this study, we also found the lumbar lordosis angle became higher after treatment, demonstrating a similar result. However, the intragroup comparison of lumbar lordosis angle showed no significant difference, it may be attributed to the small sample size of the current study, we can also suggest exercises may modify the lumbar lordosis, and then lead to pain relief and symptoms improvements.

In addition, in terms of intergroup comparison, we found that the group of core stability exercises presented with higher JOA scores and longer self-reported walking distance than conventional exercise group. We believe it indicates that core stability exercise has better effectiveness than conventional exercise for LSS. In Akhtar’s study of 120 subjects with non-specific low back pain, it illustrated that core stabilization exercise program are more effective in terms of reduction in pain, compared to routine physical therapy exercise, demonstrating a similar conclusion as our study.^17^ Core stability exercise may increase the activation of deep fibers and cross-sectional area of paravertebralmuscles^2^, facilitate the stability and coordination of lumbar spine, and subsequently result in a better clinical outcome in the treatment of low back pain.

### Limitations of the study

First, the sample size in this study was small, so some comparisons didn’t show significant differences, a large scaled clinical study may be better in evaluating the intergroup or intragroup differences. Second, both JOA and self-reported walking capacity are subjective indicators, which may be influenced by many factors. Subsequently, more studies need to be conducted in the future.

## CONCLUSION

In conclusion, we can conclude from the current study that core stability exercise presents with better efficacy than conventional exercises in treating LSS.

### Authors’ Contribution

**SJT** conceived, designed and did statistical analysis & editing of manuscript.

**WZM, YS and ZMM** did data collection and manuscript writing.

**SJT and WZM** did review and final approval of manuscript.
